# Comparison of cytotoxicity and membrane efflux pump inhibition in HepG2 cells induced by single-walled carbon nanotubes with different length and functional groups

**DOI:** 10.1038/s41598-019-43900-5

**Published:** 2019-05-17

**Authors:** Zhuoyan Shen, Jialu Wu, Yue Yu, Su Liu, Wei Jiang, Habiba Nurmamat, Bing Wu

**Affiliations:** State Key Laboratory of Pollution Control and Resource Reuse, School of the Environment, Nanjing University, Nanjing, 210023 P.R. China

**Keywords:** Environmental impact, Risk factors

## Abstract

Environmental risk of single-walled carbon nanotubes (SWCNTs) is receiving increasing attentions owing to their wide study and application. However, little is known on the influence of length and functional groups on SWCNT cytotoxicity. In this study, six types of SWCNTs with different functional groups (pristine, carboxyl group and hydroxyl group) and lengths (1–3 μm and 5–30 μm) were chosen. Cytotoxicities in human hepatoma HepG2 cells induced by these SWCNTs were compared based on cell viability, oxidative stress, plasma membrane fluidity and ABC transporter activity assays. Results showed that all the SWCNTs decreased cell viability of HepG2, increased intracellular reactive oxygen species (ROS) level, and damaged plasma membrane in a concentration-dependent manner. Long SWCNTs had stronger cytotoxic effects than short SWCNTs, which might be due to weaker aggregation for the long SWCNTs. Functionalization changed the toxic effects of the SWCNTs, and different influence was found between long SWCNTs and short SWCNTs. Moreover, the six types of SWCNTs at low concentrations changed plasma membrane fluidity, inhibited transmembrane ABC transporter (efflux pump) activity, and acted as chemosensitizer to improve the sensitivity of cells to arsenic, indicating the chemosensitive effect should be considered as toxic endpoint of SWCNTs. Comparison of different toxic endpoints among the six types of SWCNTs showed that short hydroxyl-SWCNT might be safer than other SWCNTs. This study provides insights into toxicities of SWCNTs, which is of great value for the risk assessment and application of SWCNTs.

## Introduction

Nanomaterials have been widely applied in many fields, including physical, chemical, mechanical, electrical and biomedical areas^1–3^. Among different types of nanomaterials, carbon nanotubes (CNTs) attract a great deal of attentions because of their mechanical, electrical and magnetic properties^4,5^. Therefore, there are many kinds of products emerging on the market that contain CNTs^6^. CNTs have a cylindrical structure. Their diameters are about 1–30 nm while their length can achieve tens of microns^7,8^. According to the plies of tube wall, CNTs can be divided into single-walled carbon nanotubes (SWCNTs) and multiple-walled carbon nanotubes (MWCNTs)^9^. Compared with MWCNTs, SWCNTs have some specific properties, such as less surface defects, higher capacitance, better biocompatibility and antimicrobial activity^10,11^.

Given the potential applications of SWCNTs, it is crucial to evaluate their risk to human and environment health^12^. However, compared to MWCNTs, fewer information on cytotoxicity of SWCNTs are available, and the available data are always contradictory. Some studies showed that SWCNTs exposure induced oxidative stress and inflammation^13–17^. But low cytotoxicities of SWCNTs were also reported^18,19^. Although the media depletion of nanomaterials and their interaction with the cytotoxicity assays might induce much of the contradictory results, these influence always work when high concentrations are exposed^20,21^. Furthermore, differential characteristics of SWCNTs also play important roles in the above contradictory results^22,23^. For example, some research showed that cellular uptake and responses of SWCNTs were dependent on their length^24,25^. However, Horie *et al*.^26^ thought the stability of SWCNTs suspension had more influence on cytotoxicity compared to their length. In general, chemical modification could enhance the solubility and suspension of SWCNTs in water or serum, however, there were still no consistent conclusions on how the functionalization influences cytotoxicity of SWCNTs. For example, Gutierrez-Praena *et al*.^27^ found that the carboxylic acid functionalization increased toxicity of SWCNTs. However, Sayes *et al*.^28^ proposed that the degree of sidewall functionalization would reduce the cytotoxicity of SWCNTs, and Dumortier *et al*.^29^ thought functionalized SWCNTs were non-cytotoxic. Above results show that the length and functionalization can change the cytotoxicity of SWCNTs. Thus, it is necessary to systematically determine the influence of length and functionalization on cytotoxic effects induced by SWCNTs.

Objective of this study is to compare cytotoxicity of SWCNTs with different lengths and functional groups in human hepatoma HepG2 cells. Pristine and two functionalized SWCNTs (carboxyl-SWCNTs and hydroxyl-SWCNTs) with two lengths (1–3 μm and 5–30 μm) were used to conduct cytotoxicity assays. Cell viability was chosen as the normal endpoint of cytotoxicity induced by the six SWCNTs. Oxidative stress and plasma membrane damage as two main mechanisms of SWCNT cytotoxicity were also determined^5,30^. Oxidative stress was analyzed by measuring intracellular reactive oxygen species (ROS) level. For plasma membrane damage, membrane fluidity and activity of plasma membrane ATP-binding cassette (ABC) transporter were analyzed^31,32^. This study could provide meaningful information for risk assessment and applications of SWCNTs with different lengths and functional groups.

## Materials and Methods

### Preparation and characterization of single-walled carbon nanotubes

Six types of SWCNTs including pristine long SWCNT (S-L, Product NO: XFS02), pristine short SWCNT (S-S, Product NO: XFS05), long carboxyl-SWCNT (S-L-COOH, Product NO: XFS04), short carboxyl-SWCNT (S-S-COOH, Product NO: XFS07), long hydroxyl-SWCNT (S-L-OH, Product NO: XFS03) and short hydroxyl-SWCNT (S-S-OH, Product NO: XFS06) were purchased from XFNANO Meterial Tech Co. Ltd. (Nanjing, China). Stock solutions (1000 mg/L) of the SWCNTs were prepared in deionized water and dispersed on ice by using a probe-sonicater (XO-150, Nanjing Atpio Instrument Manufacturing Co., Ltd., China) for 30 min at 60% amplitude (1:1 on/off cycle). Morphologies of the SWCNTs (1000 mg/L) were determined by using transmission electron microscopy (TEM) with a JEM-200CX electron microscope (Tokyo, Japan). Hydrodynamic radius of the SWCNTs (5 mg/L) was analyzed in culture media by dynamic light scattering (DLS) with Zetasizer Nano ZS90 (Malvern, England) at 25 °C. Three independent tests were conducted for all SWCNTs.

### Cell culture and exposure

Human hepatoma HepG2 cell line purchased from KeyGEN Biotech (Nanjing, China) was used to determine cytotoxicity of the SWCNTs. Cells were cultured in Dulbecco’s modified Eagle medium (DMEM) containing 10% fetal bovine serum (FBS) at 37 °C and 5% CO_2_. Before treatment with the SWCNTs, the cells were seeded in 96-well plates at a density of 10^4^ cells/well and incubated for 24 h.

### Assay of cell viability

Cell viability was evaluated by cell counting kit-8 (CCK-8, Dojindo Molecular Technologies, Inc., Japan). After HepG2 cells in the 96-well plate were treated with the SWCNTs (2–80 mg/L) for 24 h, 10 μL CCK-8 solution was added into the cells and incubated for 2 h at 37 °C and 5% CO_2_. Then, the cells were measured at 450 nm with a microplate reader (Synergy H1, BioTek, USA). Cell viability was calculated from the relative absorbance to control group (without SWCNT exposure).

### Assay of intracellular reactive oxygen species

Intracellular ROS level was detected by oxidant sensitive fluorescent dye 2′,7′-dichlorofluorescin diacetate (DCFH-DA, Invitrogen, USA)^33^. DCFH-DA as a non-fluorescent compound can be freely taken up by cells and oxidized to its fluorescent form (2′,7′-dichlorofluorescin, DCF), thereby indicating the level of intracellular ROS. In this study, the HepG2 cells exposed to the SWCNTs (0.2–15.0 mg/L) for 24 h were incubated with 4.87 mg/L DCFH-DA for 25 min. At the same time, cells were also exposed to ROSup (50 mg/L, KeyGEN Biotech, China) for 20 min as a positive control. For alleviating the influence from cell number loss, Hoechst 33342 (YeaSen Biotechnology, China) was used to indicate the density of cells^31^. After incubation with DCFH-DA, the microplates were washed with PBS and incubated with 5 mg/L Hoechst 33342 for 20 min. Then the cells were washed with PBS twice. The fluorescence values of DCF and Hoechst 33342 were quantified by using a microplate reader (Synergy H1, BioTek, USA). Excitation/emission wavelengths of DCF and Hoechst 33342 were 488/525 nm and 350/460 nm, respectively. Intracellular ROS level in treatment group was expressed as a percentage of that in control group (without SWCNT exposure) after the fluorescence value of DCF was divided by the fluorescence value of Hoechst 33342.

### Assay of plasma membrane fluidity

Cell membrane fluidity was measured by 4′-(trimethylammonio)-diphenylhexatrien (TMA-DPH, AAT Bioquest Inc., USA). After treatment with the SWCNTs (0.005–5 mg/L) for 24 h, the HepG2 cells in culture flask were washed by PBS. Then, the cells were re-suspended with 0.69 mg/L preheated TMA-DPH solution at 37 °C. After incubation for 20 min, the cells were washed by HEPES buffer. Fluorescence value of TMA-DPH was measured by the polarization module of microplate reader (Synergy H1, BioTek, USA) at an excitation wavelength of 485 nm and an emission wavelength of 528 nm. The relationship between fluorescence value and membrane fluidity is inverse.

### Assay of plasma membrane transporter activity

The ABC transporter as an important efflux channel of xenobiotics was chosen as target membrane transporter^34^. Its activity was determined by Calcein-AM (CAM, Dojindo, Japan). The CAM is an excellent substrate of ABC transporters and can be pumped out by P-gp and MRPs (subfamily of ABC transporters). However, when CAM is metabolized into calcein in the cells, the calcein is not the substance of ABC transporters. Then fluorescence value of CAM can indirectly indicate activity of ABC transporters^35^. In this study, after 24 h of exposure to SWCNTs (0.005–5 mg/L) or 2.69 mg/L MK571 (positive control), the HepG2 cells were incubated with 0.25 mg/L CAM for 2 h. The Hoechst 33342 was also used to normalize the fluorescence values of CAM. The excitation/emission wavelengths of CAM were 488/525 nm. Activity of ABC transporters in treatment group was expressed as a percentage of that in control group (without SWCNT exposure) after the fluorescence value of CAM was divided by the fluorescence value of Hoechst 33342.

### Analysis of chemosensitive effect of single-walled carbon nanotubes

Inhibition of ABC transporter activity can increase accumulation of substrates of ABC transporters, including environmental pollutants, which acts as the chemosensitive effect. To determine the chemosensitive effects of the SWCNTs, arsenic (As), a reported substrate of ABC transporters, was chosen as a representative environmental pollutant. After the HepG2 cells were exposed to arsenic trioxide with or without the addition of SWCNTs for 24 h, the cell viability was detected by the CCK-8 method as mentioned above. Arsenic trioxide was obtained from NSI Solution Inc. (Raleigh, USA). The 0.38 mg/L and 8 mg/L were chosen as the exposure concentrations of As and SWCNTs, respectively, since these concentrations can not induce the significant decrease in cell viability of HepG2 (*p* > 0.05).

### Statistical analysis

Three independent tests were conducted for all biological assays and each test had six replicates. All the data were shown as the mean ± standard deviation. Statistical analysis was performed by one-way analysis of variance (ANOVA) with Tukey post hoc test. A statistically significant difference was set as *p* < 0.05.

## Results

### Characteristics of single-walled carbon nanotubes

Structural parameters of the six types of SWCNTs provided by the manufacturer are shown in Table 1. Length of the long SWCNTs (5–30 μm) was about 10-times longer than the short SWCNTs (1–3 μm). Contents of carboxyl and hydroxyl groups in the functionalized SWCNTs were 2.73 wt% and 3.96 wt%, respectively. TEM images showed that diameters of the six SWCNTs were similar to those provided by the manufacturer (Fig. S1). Although the DLS measurement is a more suitable method for size determination of spherical particles, it can be still used to explain the hydrodynamic size of non-spherical materials including CNTs. In this study, hydrodynamic sizes of SWCNTs determined by DLS analysis were applied as an important evidence to indicate their conditions in culture media. Results of DLS analyses showed that the long SWCNTs had larger hydrodynamic size in cell culture media than the short SWCNTs (Table S1). Concentration of SWCNTs used in DLS measurement was 5 mg/L, which was in the range of exposure concentrations for cytotoxicity assays. Thus, results of DLS analyses could inflect the real condition of SWCNTs when they reacted with cells in culture media.

**Table 1:** Characteristics of the six types of SWCNTs used in this study.

	S-L	S-L-COOH	S-L-OH	S-S	S-S-COOH	S-S-OH
Length (μm)	5–30	5–30	5–30	1–3	1–3	1–3
Outer diameter (nm)	1–2	1–2	1–2	1–2	1–2	1–2
Inner diameter (nm)	0.8–1.6	0.8–1.6	0.8–1.6	0.8–1.6	0.8–1.6	0.8–1.6
-COOH content (wt%)		2.73			2.73	
-OH content (wt%)			3.96			3.96

### Cell viability

**Figure 1:**
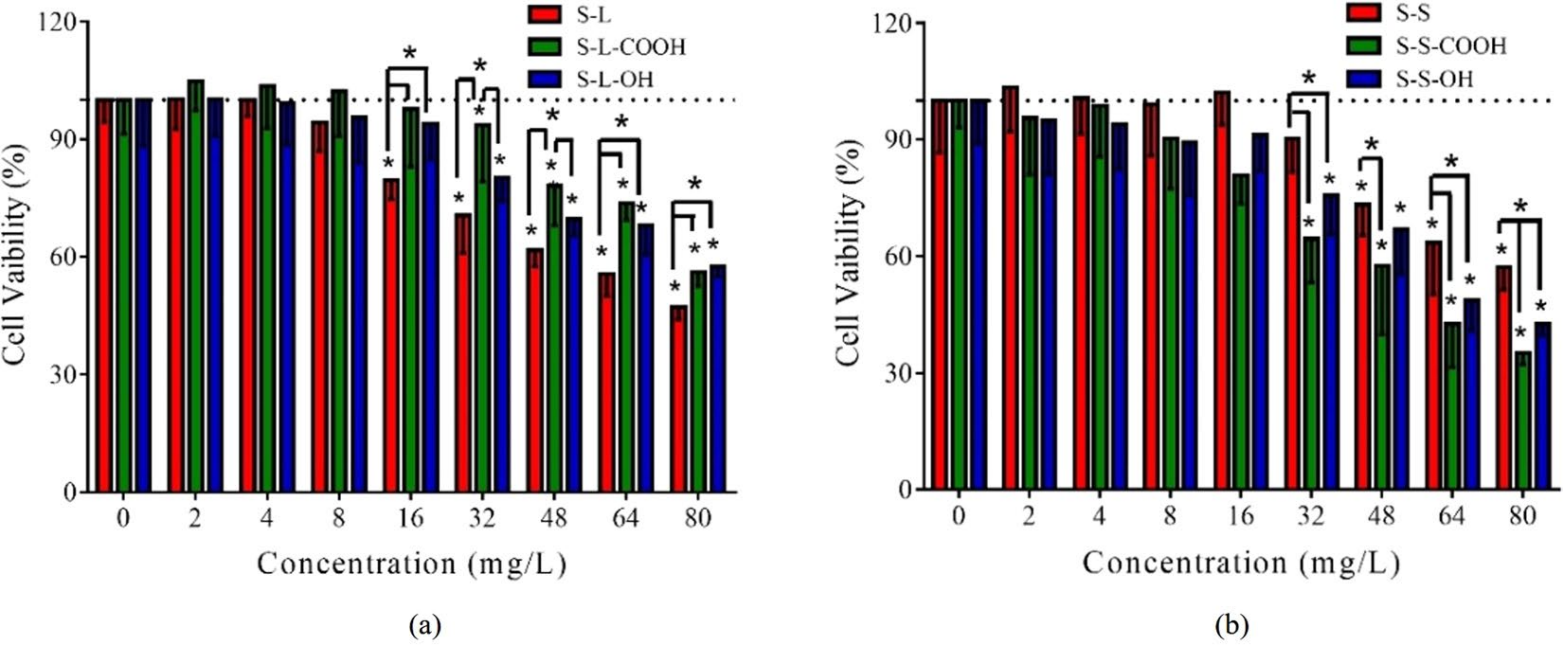
Cell viability of HepG2 treated with the SWCNTs for 24 h. (**a**) S-L, S-L-COOH and S-L-OH; (**b**) S-S, S-S-COOH and S-S-OH. All the data are shown as the mean ± standard deviation. The differences among groups were identified by one-way ANOVA followed by Tukey post hoc test. *Indicates the *p*-value < 0.05.

All the SWCNTs showed a tendency to decrease the viability of HepG2 cells in a concentration-dependent manner (Figs 1 and S2). Functionalized SWCNTs significantly decreased cell viability when exposure concentrations were ≥32 mg/L (*p* < 0.05). The lowest effective concentrations for S-L and S-S were 16 mg/L and 48 mg/L, respectively. The results are similar to previous reports of Casey *et al*.^20^. The S-L had higher cytotoxicity than functionalized long SWCNTs while that was reverse in short SWCNTs (Fig. 1).

### Generation of intracellular reactive oxygen species

**Figure 2:**
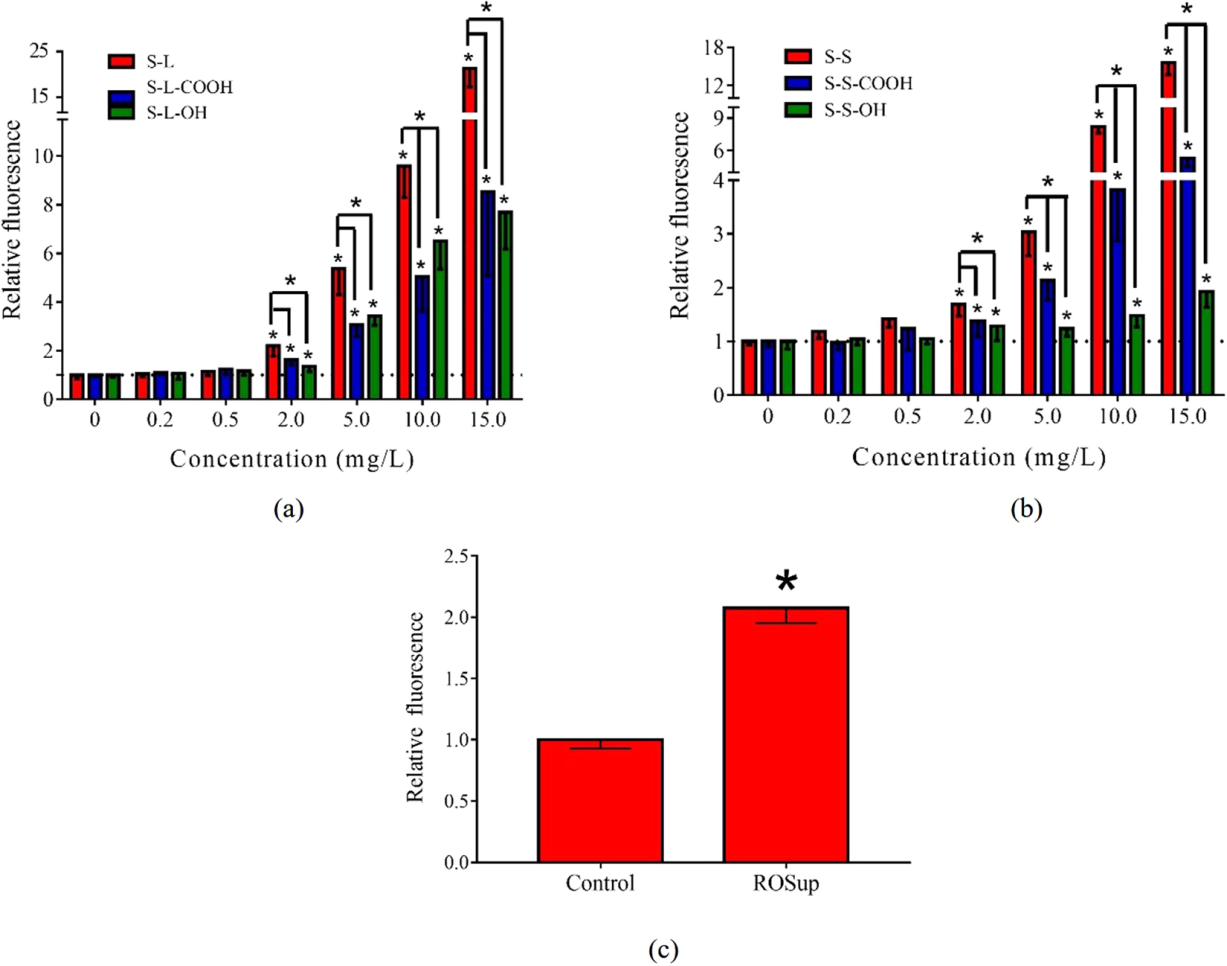
Intracellular ROS level induced by positive control and SWCNTs. Generation of ROS was determined by relative DCF fluorescence value compared to control group. (**a**) S-L, S-L-COOH and S-L-OH; (**b**) S-S, S-S-COOH and S-S-OH; (**c**) Positive control ROSup (50 mg/L, 20 min treatment). All the data are shown as the mean ± standard deviation. The differences among groups were identified by one-way ANOVA followed by Tukey post hoc test. *Indicates the *p*-value < 0.05.

Increase of intracellular ROS is one of the most important mechanisms of SWCNTs-induced cytotoxicity^30,36^. The ROSup was chosen as a positive control, which significantly increased the generation of intracellular ROS. Exposure of the six types of SWCNTs significantly increased intracellular ROS levels (*p* < 0.05) when the exposure concentration was greater than or equal to 2 mg/L (Fig. 2). Pristine SWNCTs had a stronger ability to induce ROS generation than functionalized one, no matter for long or short SWCNTs. Moreover, the cells treated with the short SWCNTs showed lower ROS generation than the long SWCNTs (Fig. S3). The S-S-OH caused the lowest intracellular ROS levels compared to other types of SWCNTs, showing an indistinctive increase with concentrations.

### Membrane fluidity

**Figure 3:**
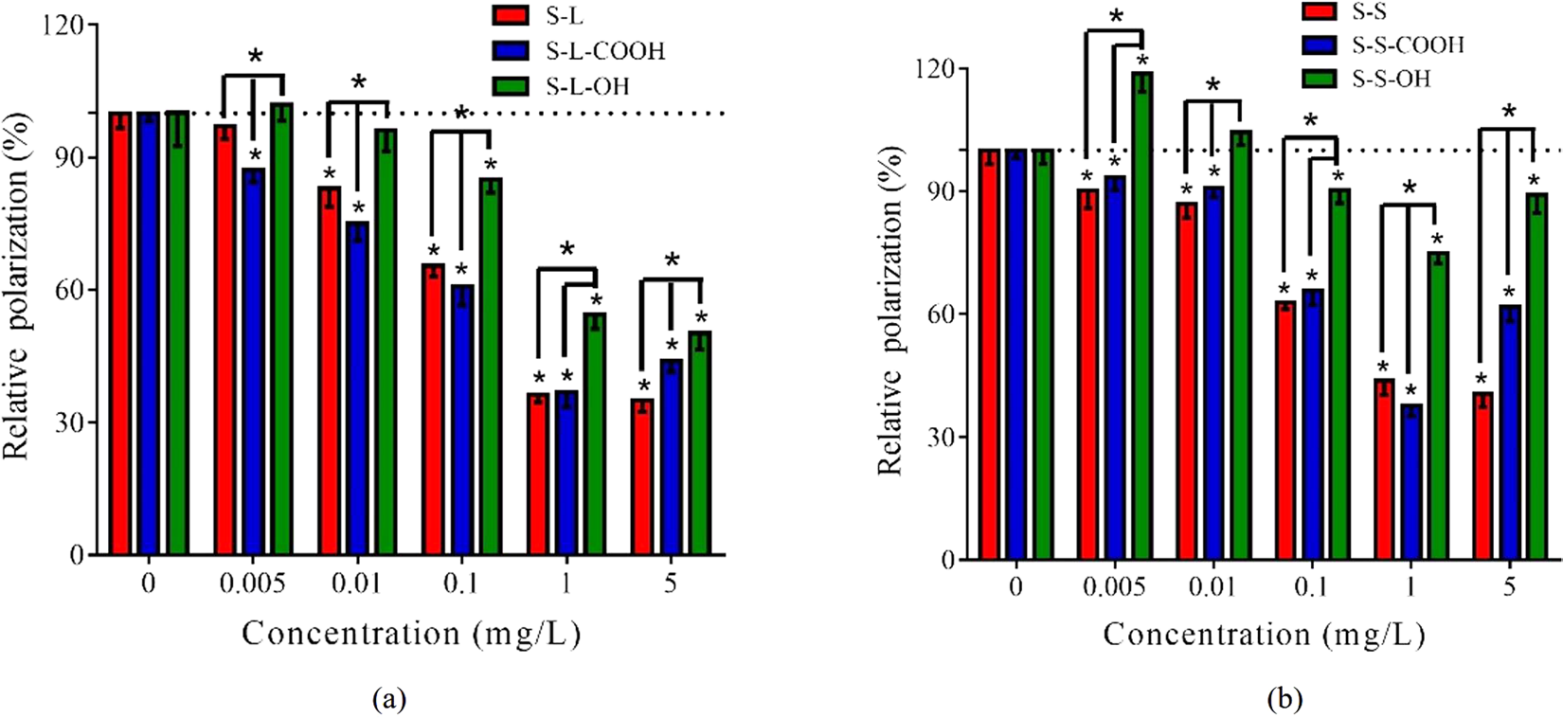
Effect of SWCNTs on cell membrane fluidity. Decrease of relative P value of TMA-DPH indicates the increase of membrane fluidity. (**a**) S-L, S-L-COOH and S-L-OH; (**b**) S-S, S-S-COOH and S-S-OH. All the data are shown as the mean ± standard deviation. The differences among groups were identified by one-way ANOVA followed by Tukey post hoc test. *Indicates the *p*-value < 0.05.

Membrane fluidity of HepG2 cells was used to investigate the influence of SWCNTs on plasma membrane^37,38^. Exposure of the six types of SWCNTs significantly decreased relative polarization (P) value of TMA-DPH, indicating increased membrane fluidity of cells (Fig. 3). The hydroxyl-SWCNTs induced significant decrease on relative P value at ≥0.1 mg/L, and the other SWCNTs induced the similar effects at ≥0.005 or 0.01 mg/L. Additionally, carboxyl-SWCNTs presented a recovery of relative P value in 5 mg/L. The similar result was observed for S-S-OH. Further, the long SWCNTs showed higher influence on plasma membrane fluidity than the short SWCNTs (Fig. S4).

### Inhibition of plasma membrane transporter activity

**Figure 4:**
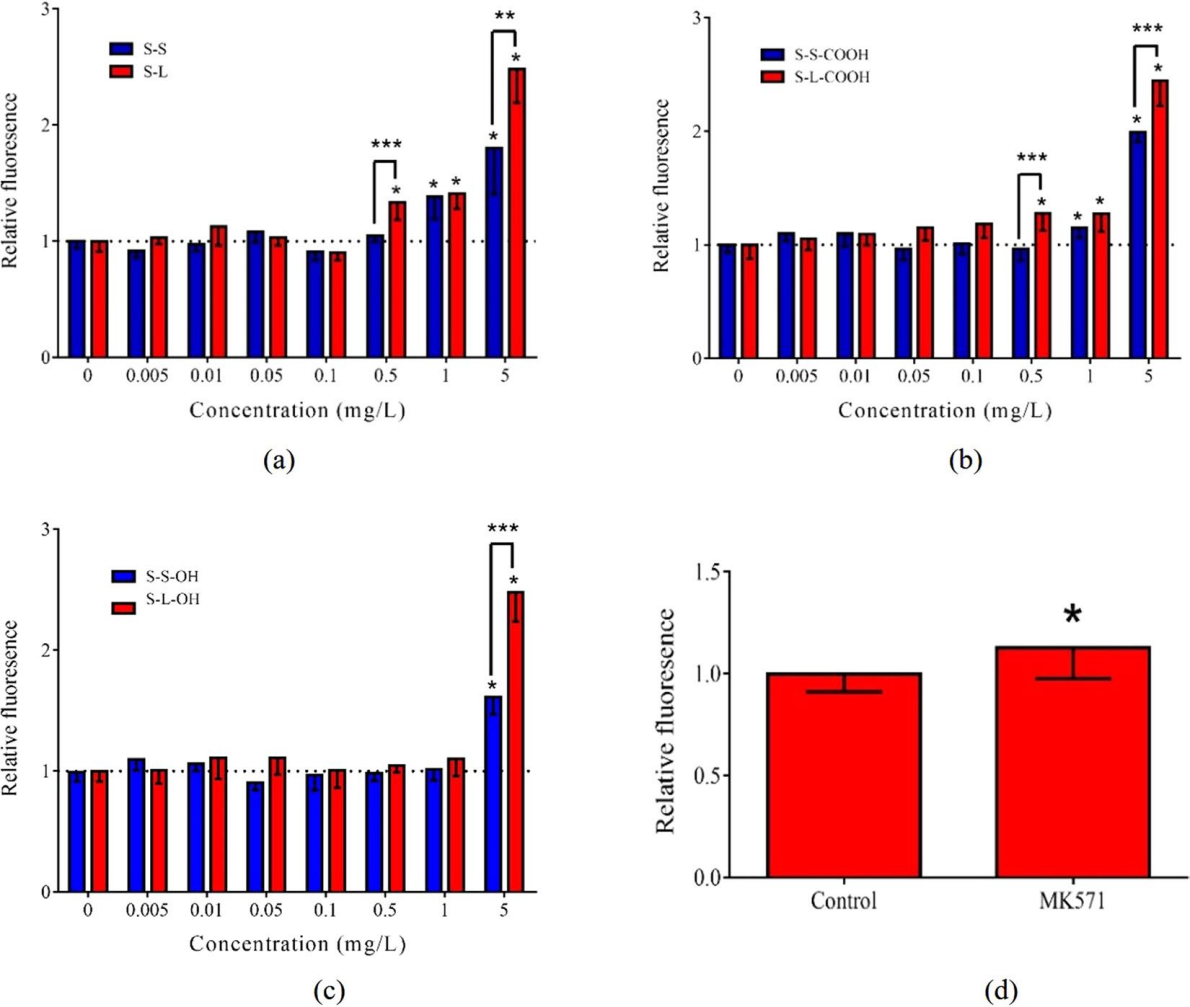
CAM accumulation induced by the SWCNTs and MK571. The relative CAM fluorescence was calculated compared to the control group. (**a**) S-L and S-S; (**b**) S-L-COOH and S-S-COOH; (**c**) S-L-OH and S-S-OH; (**d**) 2.69 mg/L MK571. All the data are shown as the mean ± standard deviation. The differences among groups were identified by one-way ANOVA followed by Tukey post hoc test. *Indicates the *p*-value < 0.05.

To further investigate the effect of SWCNTs on plasma membrane function, the membrane ABC transporter activity was analyzed (Fig. 4). The MK571 as positive control significantly inhibited ABC transporter activity in HepG2 cells (*p* < 0.05). The S-L and S-L-COOH significantly inhibited ABC transporter activity at ≥0.5 mg/L, while S-S and S-S-COOH showed significant effect at ≥1 mg/L. The lowest effective concentration for SWCNTs with hydroxyl group was 5 mg/L. Furthermore, the long SWCNTs had more notable influence on inhibition of ABC transporter activity than the short SWCNTs.

**Figure 5:**
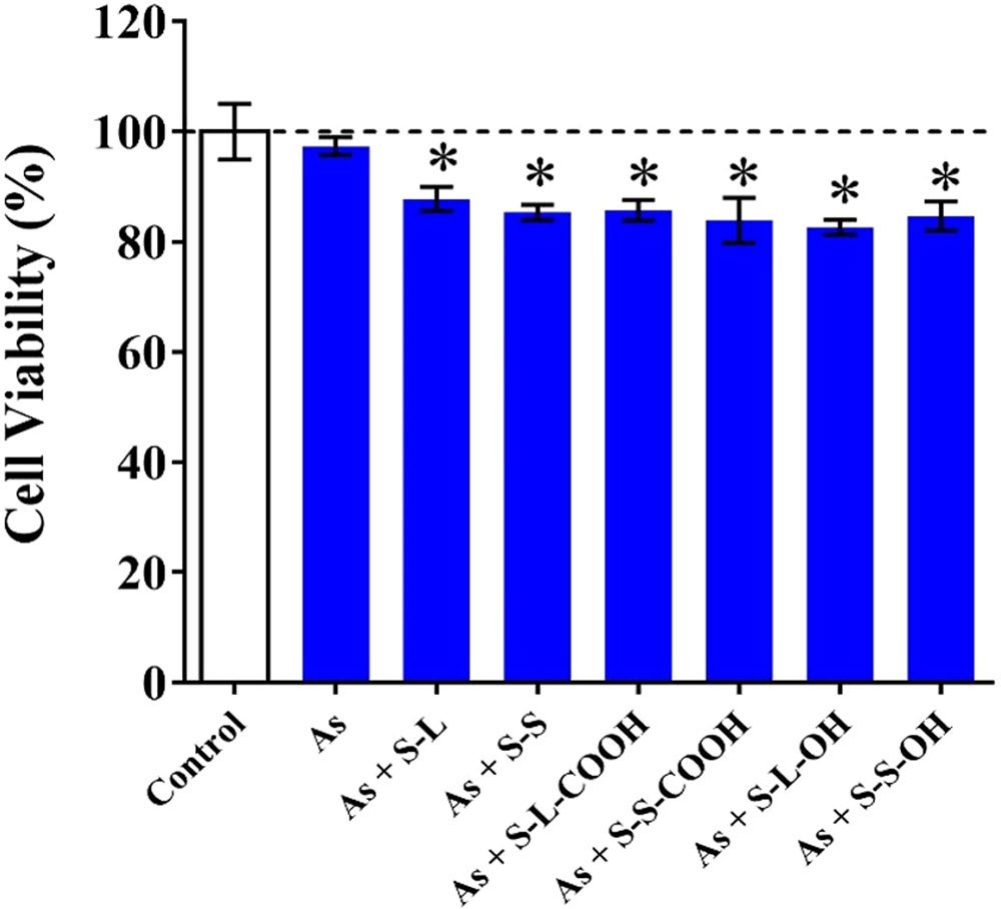
Combined effect of SWCNTs (8 mg/L) and As (0.38 mg/L) on cell viability of HepG2. All the data are shown as the mean ± standard deviation. The differences among groups were identified by one-way ANOVA followed by Tukey post hoc test. *Indicates the p-value < 0.05.

The As was chosen as a model pollutant to evaluate potential chemosensitive effect of SWCNTs due to the inhibition of ABC transporter activity. Glutathione adduct of As is one of the special substrate of MRPs, also named ABCCs, which are the member of ABC transporter family. All the SWCNTs significantly decreased cell viability induced by As exposure (Fig. 5), indicating the chemosensitive effect of the SWCNTs.

## Discussion

SWCNTs have been widely studied and applied in many areas. Thus, their potential risks to human and environment health are receiving increasing attentions. However, it is still unclear how length and functional groups influence SWCNT toxicity. This study compared the cytotoxicities induced by SWCNTs with different lengths and functional groups by assays of cell viability, intracellular ROS level and plasma membrane damage.

Previous literatures showed that SWCNTs could inhibit cell viability of human ocular cells and human keratinocyte cells^39,40^. Similar to these studies, this study also found that the six types of SWCNTs decreased cell viability of HepG2 in a concentration-dependent manner (Fig. 1). The carboxyl and hydroxyl groups decreased the cytotoxicity of long SWCNTs, but increased the toxic effects of short SWCNTs. Some literatures showed that the modification could increase the cytotoxicity of short SWCNTs^18,27,41,42^. However, this study found that the influence of functional groups was differentiated by length of SWCNTs, which is not mentioned in previous literatures.

Mechanisms of SWCNTs-induced toxicity are focused on the oxidative stress and the plasma membrane damage. This study found that the six types of SWCNTs increased intracellular ROS levels in HepG2 cells. Intracellular ROS generation and oxidative stress induced by the SWCNTs could lead to inflammation, organelle stress, even apoptosis or necrosis^30,43^. Thus, intracellular ROS generation might be more sensitive than cell viability assay. In this study, the six types of SWCNTs indeed induced increase of ROS level at ≥2 mg/L, which were lower than the lowest effective dose of cell viability. Functionalized SWCNTs presented lower effect on ROS generation than pristine SWCNTs. However, the influences of functionalization on cytotoxic effect of SWCNTs reported in the published literatures are contradictory. Some studies found that chemical modification increased the cytotoxic effect of SWCNTs^27,42^, while other research were in consistence with our result^28,29,44^. The contradictory might be due to the complicated interaction between cells and SWCNTs. First, it is widely believed that the functional groups, like carboxyl and hydroxyl groups, could increase the solubility of nanomaterials, increasing their interaction with cells^45,46^. On the other hand, carboxyl and hydroxyl groups increase the negative charged surface of SWCNTs, which make SWCNTs more difficultly interact with cell membrane^47^. Additionally, differences on cell lines used in the different studies might also influence the cytotoxic effects of SWCNTs with different functional groups. For length of SWCNTs, the long SWCNTs stimulated more generation of ROS than the short SWCNTs, which was similar to the report of Wang *et al*.^48^. The long SWCNTs have higher aspect ratio and weaker aggregation than the short SWCNTs^24,38^, which might be the reason of higher ROS generation. Overall, this study indicated the long and pristine SWCNTs more easily induced the intracellular ROS generation.

Influences of functional groups on the long SWCNT - induced toxicity obtained from cell viability and ROS generation assays were consistent. But for short SWCNTs, different influences were obtained. Pristine short SWCNTs could induce more intracellular ROS than functionalized short SWCNTs at ≤15 mg/L (Fig. 2) but reversed between 32–80 mg/L based on CCK-8 assay. The short SWCNTs had stronger aggregation than the long SWCNTs^49^. Thus, the short SWCNTs tend to aggregate themselves in cell culture media, leading to lower cytotoxicity. The chemical modification with carboxyl and hydroxyl groups can prevent aggregation and enhance biocompatibility of CNTs^46,50^. Therefore, it was possible that the S-S showed slight cytotoxicity at high exposure concentrations (32–80 mg/L) by stronger aggregation which was not happened in the functionalized short SWCNTs. However, for low exposure concentrations, the aggregation was weak. Then the negative charges of functional groups played main roles, which made SWNCTs more difficultly interact with the cells, leading to lower generation of intracellular ROS than pristine short SWCNTs.

Interference on plasma membrane was also commonly used to assess the cytotoxic effects of CNTs^30^. The size and shape of SWCNTs make them easily interact with cell surface. Thus, the damages in plasma membrane induced by SWCNTs might more sensitive than cell viability and intracellular ROS generation. In this study, all the SWCNTs at 0.005–5 mg/L significantly enhanced membrane fluidity (Fig. 3). The effective doses in membrane fluidity were indeed lower than above two toxic endpoints. The enhancement on membrane fluidity was also found in a research of SWCNTs on bacteria, which suggested that SWCNTs might increase membrane fluidity through fluidizing the cytoplasmic membrane or destroying bacterial membranes^38^. This study also found that the long SWCNTs showed stronger influence than the short SWCNTs (Fig. 3), which might be due to that the long SWCNTs had higher contact area and weaker aggregation than the short SWCNTs^49^.

Changes in plasma membrane fluidity can disrupt the lipid bilayer, which further affect functions of biomolecules residing in the plasma membrane^51^. This study found that low levels of SWCNTs significantly inhibited the activity of ABC transporter (Fig. 4). Our previous studies on MWCNTs with different length and functional groups also found the similar results^32^. The MWCNTs could inhibit ABC transporter activity by damaging membrane structure and functions, including membrane fluidity. In this study, the hydroxyl-SWCNTs induced the lowest inhibiting effects, followed by the pristine SWCNTs and carboxyl-SWCNTs. The differences were similar to those in the membrane fluidity assay. The uniformity of effective doses indicates the changes in ABC transporter activity might be due to the disorder in membrane fluidity. Moreover, this study found the SWCNTs with non-toxic concentration significantly increased cytotoxicity induced by As, indicating that the inhibition of ABC transporter could increase accumulation of its substrate^31,32^. Thus, the chemosensitive effect should be considered as toxic endpoint of SWCNTs.

## Conclusions

The six types of SWCNTs could decrease cell viability, induce generation of oxidative stress and damage plasma membrane functions in HepG2 cells. The long SWCNTs had stronger cytotoxic effects than the short SWCNTs. Functionalization changed the adverse effects of SWCNTs, but different influences were found between the long and short SWCNTs. It was notable that the SWCNTs at low concentrations changed plasma membrane fluidity and inhibited activity of ABC transporter, which make SWCNTs act as chemosensitizer to improve the sensitivity of cells to environmental pollutants. Comprehensive comparison of different toxic endpoints among the six types of SWCNTs showed that S-S-OH might be safer than the other SWCNTs. This study provides basic information on different toxicity of SWCNTs with different length and functional groups, which is useful for the risk assessment of SWCNTs.

## Supplementary information

Supporting Information
