# Supplementary material for: Comparison of cytotoxicity and membrane efflux pump inhibition in HepG2 cells induced by single-walled carbon nanotubes with different length and functional groups
Source: Sci Rep. 2019 May 17;9:7557. doi: 10.1038/s41598-019-43900-5 (PMC6525162; doi:10.1038/s41598-019-43900-5)
Supplement: Supplementary file 1 — Supporting Information [file 41598_2019_43900_MOESM1_ESM.pdf]

Supporting Information for

Comparison of cytotoxicity and membrane efflux pump inhibition in HepG2 cells induced by single-walled carbon nanotubes with different length and functional groups

Zhuoyan Shen, Jialu Wu, Yue Yu, Su Liu, Wei Jiang, Habiba Nurmamat, Bing Wu*

State Key Laboratory of Pollution Control and Resource Reuse, School of the Environment, Nanjing University, Nanjing, 210023, P.R. China

* Corresponding author

E-mail: bwu@nju.edu.cn (B.Wu)

Postal address: NO 163 Xianlin Avenue, Nanjing, 210023, China

Table S1. Z-average size of six SWCNTs used in this study

	S-L	S-L-COOH	S-L-OH	S-S	S-S-COOH	S-S-OH
Z-average size (nm)	375.7	491.3	555.6	201.9	156.9	178.2

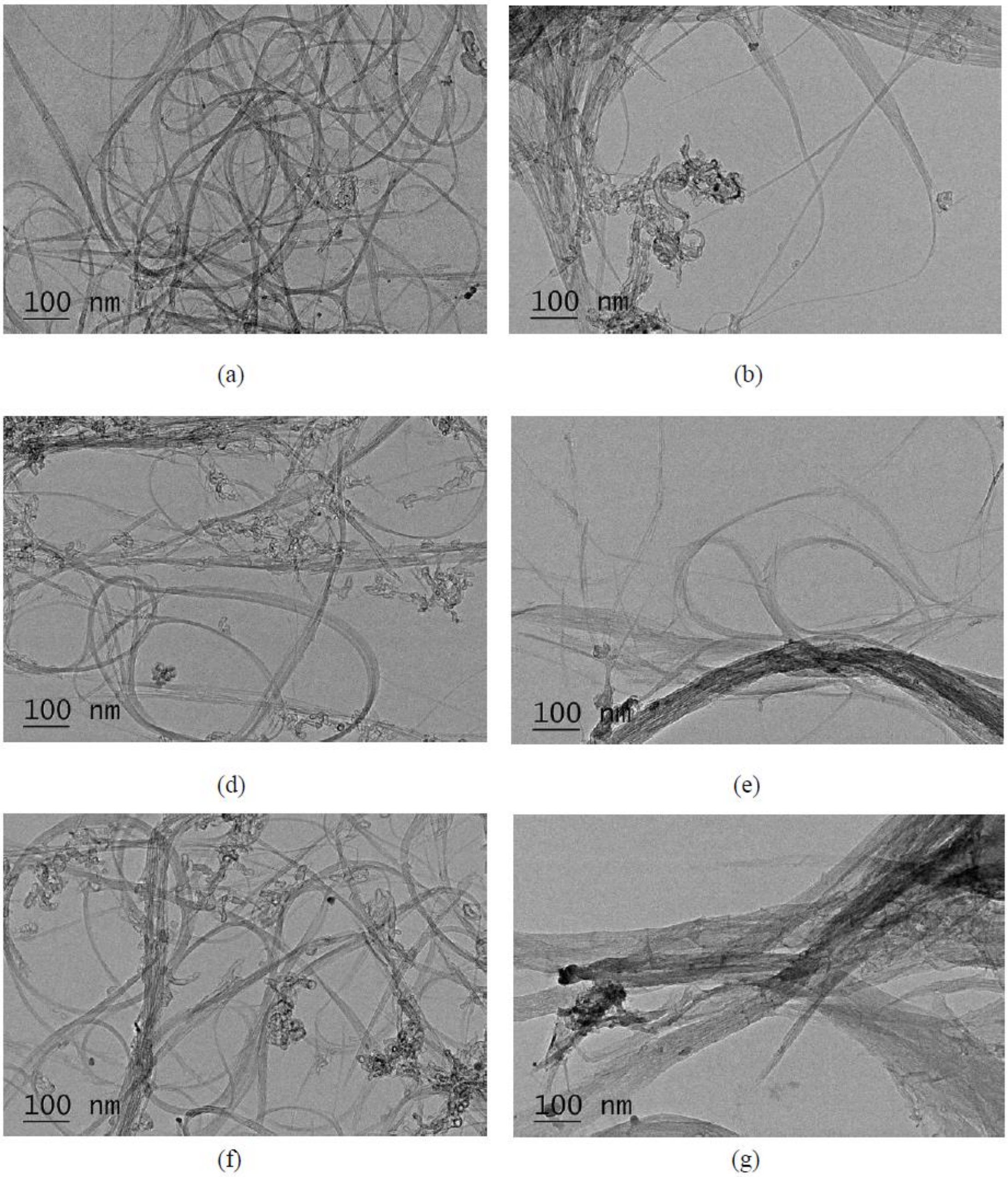

Fig. S1 TEM images of the six SWCNTs used in this study. (a) S-L; (b) S-S; (c) S-L-COOH; (d) S- S-COOH; (e) S-L-OH; (f) S-S-OH

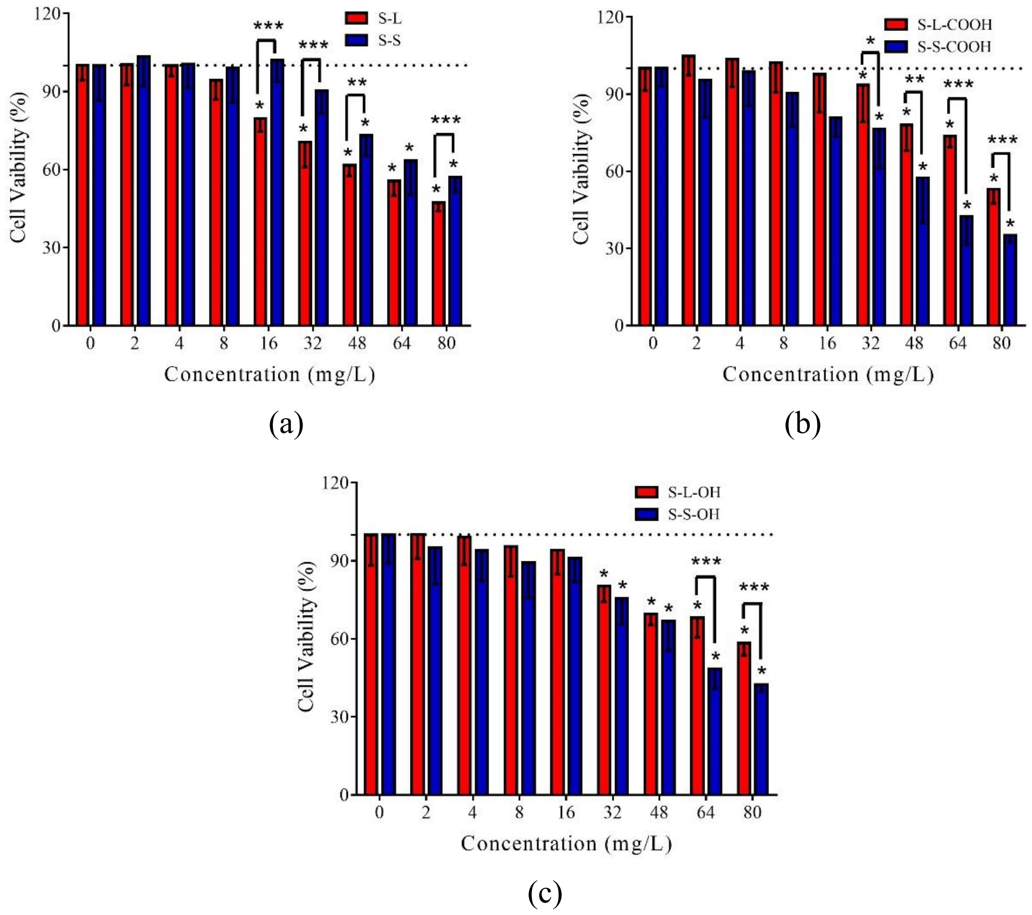

Fig. S2 Cell viability of HepG2 cells treated with the SWCNTs for 24 h. (a) S-L and S-S; (b) S-L-COOH and S-S-COOH; (c) S-L-OH and S-S-OH. All the data are shown as the mean ± standard deviation. The differences among groups were identified by one-way ANOVA followed by Tukey post hoc test. * indicates the p-value < 0.05.

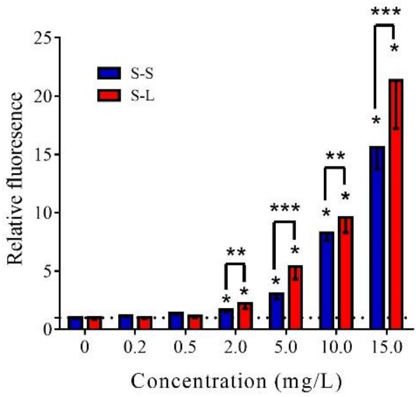

(a)

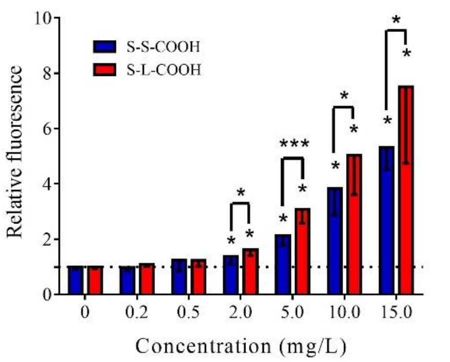

(b)

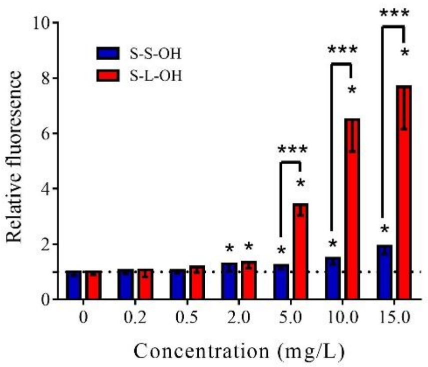

(c)

Fig. S3 Intracellular ROS level induced by the SWCNTs. (a) S-L and S-S; (b) S-L-COOH and S-S-COOH; (c) S-L-OH and S-S-OH. All the data are shown as the mean ± standard deviation. The differences among groups were identified by one-way ANOVA followed by Tukey post hoc test. * indicates the p-value < 0.05.

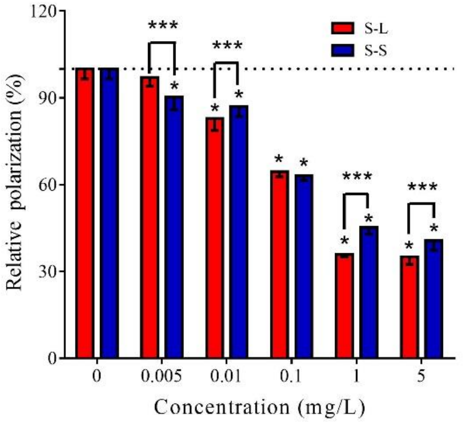

(a)

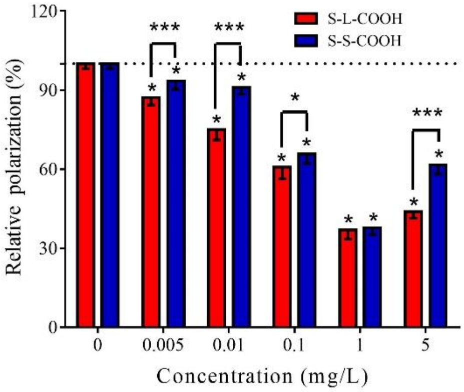

(b)

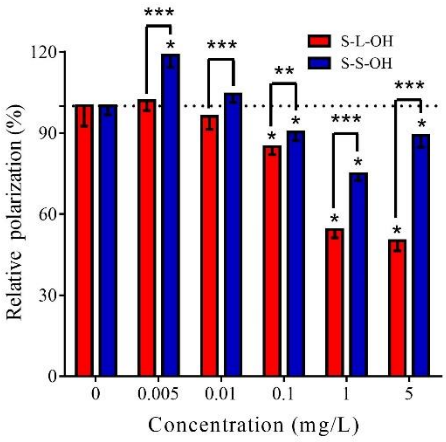

(c)

Fig. S4 Effect of SWCNTs on cell membrane fluidity. Decrease of relative P value of TMA-DPH indicates the increase of membrane fluidity. (a) S-L and S-S; (b) S-L-COOH and S-S-COOH; (c) S-L-OH and S-S-OH. All the data are shown as the mean ± standard deviation. The differences among groups were identified by one-way ANOVA followed by Tukey post hoc test. * indicates the p-value < 0.05.
